# Arrhythmogenic Right Ventricular Cardiomyopathy/Dysplasia (ARVC/D) - What We Have Learned after 40 Years of the Diagnosis of This Clinical Entity

**DOI:** 10.5935/abc.20180266

**Published:** 2019-01

**Authors:** Jorge Elias Neto, Joelci Tonet, Robert Frank, Guy Fontaine

**Affiliations:** 1 Vitória Apart Hospital - Serviço de Eletrofisiologia, Serra, ES - Brazil; 2 ‘Unité de Rythmologie de l'Institut de Cardiologie de l'Hôpital Pitié-Salpêtrière, Paris - France

**Keywords:** Arrhythmogenic Right Ventricular Dysplasia/physiopathology, Arrhythmias, Cardiac/diagnostic imaging, Catheter Ablation, Defibrillators, Implantable, Magnetic Resonance Imaging

## Abstract

Arrhythmogenic right ventricular cardiomyopathy/dysplasia (ARVC/D) was initially recognized as a clinical entity by Fontaine and Marcus, who evaluated a group of patients with ventricular tachyarrhythmia from a structurally impaired right ventricle (RV). Since then, there have been significant advances in the understanding of the pathophysiology, manifestation and clinical progression, and prognosis of the pathology. The identification of genetic mutations impairing cardiac desmosomes led to the inclusion of this entity in the classification of cardiomyopathies. Classically, ARVC/D is an inherited disease characterized by ventricular arrhythmias, right and / or left ventricular dysfunction; and fibro-fatty substitution of cardiomyocytes; its identification can often be challenging, due to heterogeneous clinical presentation, highly variable intra- and inter-family expressiveness, and incomplete penetrance.

In the absence of a gold standard that allows the diagnosis of ARVC/D, several diagnostic categories were combined and recently reviewed for a higher diagnostic sensitivity, without compromising the specificity. The finding that electrical abnormalities, particularly ventricular arrhythmias, usually precede structural abnormalities is extremely important for risk stratification in positive genetic members. Among the complementary exams, cardiac magnetic resonance imaging (CMR) allows the early diagnosis of left ventricular impairment, even before morpho-functional abnormalities. Risk stratification remains a major clinical challenge, and antiarrhythmic drugs, catheter ablation and implantable cardioverter defibrillator are the currently available therapeutic tools. The disqualification of the sport prevents cases of sudden death because the effort can trigger not only the electrical instability, but also the onset and progression of the disease.

## Introduction

ARVC/D is an inherited disease of the heart muscle that predominantly affects the right ventricle (RV). It is characterized by the progressive loss of the right ventricular myocardial tissue and its replacement for fibrous and fatty tissue.^1^^,^^2^

Originally described by Fontaine and Marcus in 1982, ARVC/D is one of the leading causes of sudden arrhythmic death (SAD) in young people and athletes.^3^ Recently, there have been substantial advances in the understanding of its pathogenesis, clinical manifestations and long-term progression.^4^

The disease was initially referred to as dysplasia because it was thought to be a congenital defect of the RV myocardial development. The subsequent finding that the disease is caused by a genetic defect in cardiac desmosomes allowed its description as cardiomyopathy, and its inclusion in the classification of cardiomyopathies by the American Heart Association (AHA).^4^^-^^7^

### Etiopathogenesis

#### Histopathological characteristics

The characteristic histopathological finding of ARVC/D is the progressive loss of RV myocardial tissue that is replaced by fibrous and fatty tissue.

The presence of irregular mononuclear inflammatory infiltrate (predominantly lymphocytic) is common, suggesting that the process may have an immunological mediation.^8^ It has been proposed that the inflammatory infiltrate can extend the lesion to previously healthy regions, a process associated with worsening of the electrocardiographic abnormalities with consequent increase in symptomatic arrhythmias. This type of progression may be confused with acute myocarditis.^8^

Contrary to what was observed in several forms of heart disease, in which there was a predominance of subendocardial muscle involvement, in the ARVC/D the greatest impairment is evident in the subepicardial region of the RV free wall. In addition, segments of the RV free wall that experience the greatest mechanical stress during the cardiac cycle are more impaired. In general, the trabecular muscles of the RV endocardial region and the interventricular septum (relevant aspect when differentiating from sarcoidosis) are spared. When the left ventricle (LV) is involved, myocardial degeneration and fibrosis are more visible in the subepicardium and in the middle myocardium of the lateral wall.^1^^,^^8^

In the typical ARVC/D form, the LV is affected to a lesser degree than the RV; however, there are variants of the disease characterized by equivalent or even predominant involvement of the LV.^1^^,^^4^^,^^5^

#### Genetic and molecular characteristics

In most cases, ARVC/D is an inherited disease with an autosomal dominant pattern, with variable penetrance and expressivity. Among the probands diagnosed with the disease, a screening of first-degree relatives allows the identification of the presence of genetic mutations in approximately 50% of cases, regardless of gender. In a small number of cases, ARVC/D has an autosomal recessive pattern as part of a cardiocutaneous syndrome (Naxos disease or Carvajal syndrome), characterized by woolly hair and palmoplantar keratoderma.^4^^,^^6^

As observed in other familial diseases, there is a high degree of heterogeneity in ARVC/D. To date, mutations in more than 12 genes have been identified as causing ARVC/D, although many of these genes are also responsible for other diseases.^9^

Other patients with ARVC/D may have genetic abnormalities whose mutations have not been identified yet. These mutations may be inherited from family members or be the result of a new mutation.^9^

An individual who has a ARVC/D mutation may or may not develop signs and symptoms of the disease. Recent studies suggest the presence of one or more additional genetic abnormalities in a single gene class, such as plakophilin-2 (PKP2), for example, which may determine when a mutation-carrier individual may be clinically affected by the disease.^10^^-^^12^

Mutations may be in desmosomal and non-desmosomal genes. These mutations can be found and registered at the electronic address: https://doi.org/10.1002/humu.22765 o.^13^

#### Importance and Limitations of Genetic Testing

Genetic testing can be useful to determine the diagnosis in an individual suspected of having ARVC/D, and to identify relatives who do not have signs and symptoms of ARVC/D but who are carriers of the genetic defect. If an abnormal gene is identified in a proband and not in family members, it is unlikely that these members will have the disease based on this genetic abnormality.^4^^,^^5^^,^^10^^,^^11^ However, there are several observations that limit the analysis and the use of the genetic test in ARVC/D:

- The proband may have a second unidentifiable genetic defect.
- The gene most commonly related to the manifestation of signs and symptoms of ARVC/D is that of *PKP2*. However, this genetic abnormality may require a second mutation in that same gene or in another desmosomal gene for the disease to manifest itself. That is, the simple identification of the gene cannot define whether it is the cause of the disease.
- Not being able to identify all the genes associated with the pathology, as well as the existence of combined mutations, make ARVC/D a genetically complex disease, which makes family counseling difficult.^11^

Periodic examinations should be performed on all individuals with genetic abnormalities for ARVC/D. It is recommended that cardiac evaluation be started between 10 and 12 years of age, because the manifestation of the disease before this age is rare. It is suggested that the tests include electrocardiogram, high resolution electrocardiogram (ECG-HR), echocardiogram and, if possible, CMR and 24-hour Holter. It is recommended that this evaluation is repeated every 2 years, between 10 and 20 years of age, and every 5 years after 20 years of age. The evaluation may be interrupted between 50 and 60 years of age because the presentation of the disease in this age group is uncommon.

An additional advantage of the genetic test lies in the aid of the differential diagnosis, as in the case of cardiac sarcoidosis, which can mimic the signs and symptoms of ARVC/D.

In addition, recent molecular biology studies have again put into perspective the debate about a possible pathogenic link between ARVC/D and Brugada syndrome (BS).^4^^,^^12^^,^^13^

### Clinical presentation and natural history

#### Epidemiology

ARVC/D has an age-dependent penetrance and is typically manifested between the 3^rd^ and 5^th^ decade of life in the form of episodes of ventricular arrhythmias that may progress to SAD. The estimated prevalence ranges from 1:2,000 to 1:5,000, with a predominance in the Caucasian population and in participants in strenuous exercise or competitive sports.^1^^,^^6^^,^^7^

Despite its low prevalence, ARVC/D accounts for approximately 5% to 20% of SAD cases in young people. The occurrence of ARVC/D in individuals younger than 12 years of age, or older than 60 years is extremely rare.^7^^,^^14^^,^^15^

The disease is more malignant in men than in women, a finding that can be explained by a direct influence of sex hormones on the mechanisms involved in the phenotypic expression of the disease, or by differences in the amount and intensity of physical effort.^5^

#### Clinical and natural history

The natural history of ARVC/D, in its classic form (dominant RV), can be classified into 4 distinct phases, according to the progression of structural alterations and clinical symptomatology:

- **Occult phase:** this is the subclinical phase, in which the patient remains asymptomatic and with discrete structural abnormalities in the RV or without them. At this stage, SAD may be the first manifestation of the disease.
- **Arrhythmic phase:** the patient has palpitations, syncope and, generally, symptomatic ventricular arrhythmias originating in the RV, triggered by physical effort. Arrhythmias may range from isolated ventricular ectopies (non-sustained ventricular tachycardia) (NSVT) with left bundle-branch block morphology (LBBB) until reaching SAD episodes due to ventricular fibrillation.
- **Right ventricular failure**: The progressive replacement of myocardial tissue with fibro-fatty tissue leads to progressive impairment of RV function, which can lead to heart failure.
- **Biventricular failure:** In an advanced stage of the disease, the interventricular septum is involved causing congestive heart failure. At this stage, mural thrombosis may occur, especially in aneurysms that form in the RV or in the presence of atrial fibrillation. The phenotype may mimic advanced dilated cardiomyopathy, hindering the differential diagnosis in the more advanced stages of the disease.^16^

Recently, Calkins et al.^6^ reported the clinical follow-up of a cohort of 102 patients diagnosed with ARVC/D after 50 years of age. The authors observed that, although SVT is also frequent in this age group, the incidence of syncope, typical electrocardiographic changes, ventricular ectopy to the Holter, and pathogenic mutation were less prevalent than in the younger age groups.^6^ A later manifestation of ARVC/D does not translate into a better prognosis of survival free of high-risk arrhythmic events.^14^

### Clinical diagnosis

In general, the diagnosis of ARVC/D should be considered in any young or middle-aged individual presenting: (1) frequent ventricular ectopies; (2) ventricular tachycardia with LBBB morphology with superior or multiple QRS morphologies; and (3) SAD. This hypothesis is reinforced in cases of arrhythmic events that occur during exercise in individuals with inverted T-waves in right precordial leads.^7^

Although these clinical indicators lead to the diagnostic hypothesis, the definitive diagnosis of ARVC/D remains a challenge because it is a disease with a low prevalence that lacks a single conclusive diagnostic test.^17^

To standardize the clinical diagnosis of ARVC/D, in 1994 an international task force (TFC 94) proposed guidelines in the form of a qualitative scoring system with major and minor criteria.^1^^,^^4^ In 2010, the task force reviewed the guidelines for improving diagnostic sensitivity, especially for family members (TFC 2010),^18^ providing quantitative criteria for the diagnosis of RV abnormalities and aggregating molecular genetic criteria (Table 1).^4^^,^^16^

**Table 1:** Task Force Criteria reviewed

1. Structural changes and global or regional dysfunction
**Major criteria**
• Two-dimensional echocardiogram
□ Akinesia, dyskinesia or regional RV aneurysm associated with one of the following diastole measures:
– PLAX RVOT ≥ 32 mm (PLAX / BSA ≥ 19 mm/m^2^) or
– PSAX RVOT ≥ 36 mm (PSAX/BSA ≥ 21 mm/m^2^) or
– Fractional area change ≤ 33%
• CMR
□ Akinesia or regional RV dyskinesia or dyssynchronism of RV contraction associated with one of the following measures:
– RV EDV/BSA ≥ 110 mL/m^2^ (male) or ≥ 100 mL/m^2^ (fem.)
– RV ejection fraction ≤ 40%
• Right ventriculography
□ Akinesia, dyskinesia or RV aneurysm
**Minor criteria**
• Two-dimensional echocardiogram
□ Akinesia, RV dyskinesia or dissyncronism of RV contraction and one of the measures of diastolic function below:
– PLAX RVOT ≥ 29 to < 32 mm (PLAX/BSA ≥ 16 to < 19 mm/m^2^) or
– PSAX RVOT ≥ 32 to < 36 mm (PSAX/BSA ≥ 18 to < 21 mm/m^2^) or
– Fractional area change> 33% ≤40%
• CMR
□ Akinesia or regional RV dyskinesia or dyssynchronism of RV contraction and one of the following measures:
– RV EDV/BSA ≥ 100 to 110 mL/m^2^ (male) or ≥ 90 to 100 mL/m^2^ (fem.)
– RV ejection fraction > 40 to ≤ 45%
**2. Tissue aspects**
**Major criteria**
• Residual myocyte count <60% by morphometric analysis (or <50%, if estimated), with fibrous RV free wall replacement in ≥1 sample, with or without fat replacement of endomyocardial biopsy tissue
**Minor criteria**
• Residual myocyte count of 60% to 75% by morphometric analysis (or 50% to 65% if estimated), with fibrous RV free wall replacement in ≥1 sample, with or without fat replacement of endomyocardial biopsy tissue
**3. Repolarization abnormalities**
**Major criteria**
• Inverted T waves in the right precordial vessels (V1, V2, and V3) or extending beyond V3 in individuals > 14 years of age (in the absence of RBBB-QRS ≥ 120 ms)
**Minor criteria**
• Inverted T waves in V1 and V2 in ind. > 14 years of age (in the absence of RBBB)
• Inverted T waves in V1, V2, V3, AND V4 in ind. > 14 years, in the presence of RBBB
**4. Depolarization / conduction abnormalities**
**Major criteria**
• Epsilon wave (reproducible low amplitude signals between the end of the QRS and the beginning of the T wave) in the right precordial leads (V1 - V3)
**Minor criteria**
• Late potentials on the ECG-HR in ≥ 1 of the 3 parameters in the absence of QRSd ≥ 110 msec in the 12-lead ECG:
□ Filtered QRS duration (fQRS) ≥ 114 msec
□ Duration of terminal QRS < 40 micro V ≥ 38 ms
□ Root-mean-square voltage of terminal 40 ms ≤ 20 micro V
• Duration of the final QRS portion ≥ 55 ms (measurement of nadir from S wave to end of ventricular depolarization - including R’) in V 1, V 2 or V 3
**5. Arrhythmias**
**Major criteria**
• Non-sustained or sustained VT with RBBB type morphology and upper axis
**Minor criteria**
• Non-sustained or sustained VT with RVOT morphology (LBBB type morphology and lower or indeterminate axis) > 500E vs/24h - 24h Holter
**6. Family History**
**Major criteria**
• ARVC/D in first-degree relative who meets TFC2010 criteria
• ARVC/D pathologically confirmed in first degree relative (autopsy or biopsy)
• Identification of pathogenic mutation classified as associated or probably associated with ARVC/D in the patient under evaluation
**Minor criteria**
• History of ARVC/D in first degree relatives
• History of ARVC/D in a first-degree relative for whom it is not possible to determine whether it meets TFC criteria
• Sudden premature death (< 35 years of age) with suspected ARVC/D in first degree relative
• ARVC/D confirmed pathologically or through TFC in second degree relative

Adapted from Pinamonti et al., 2014.^16^ ARVC/D: right ventricular arrhythmogenic cardiopathy/dysplasia; BSA: body surface area; CMR: cardiac magnetic resonance; ECG: electrocardiogram; EDV: end-diastolic volume; RBBB: right bundle Branch block; LBBB: left bundle Branch block; PLAX: parasternal long axis; PSAX: parasternal short axis; RV right ventricle; RVOT: right ventricular outflow tract; ECG-HR: high resolution electrocardiogram; Ventricular tachycardia; TFC task force criteria.

Although it is the current gold standard, TFC 2010 does not apply to the predominant forms of involvement of the left chambers^19^ that may be included in future reviews.^4^^,^^20^

Patients are diagnosed as having ARVC/D if they present a total of 4 points considering that the major criterion value is 2 points; and the minor criterion, 1 point. Patients who reach the “3-point” score are classified as probable ARVC/D carriers, while those with 1 or 2 points are classified as not meeting the criteria for ARVC/D.^6^^,^^18^

The initial evaluation consists of non-invasive examinations (ECG, ECG-HR, echocardiogram (ECHO) and/or CMR, 24-hour Holter and genetic analysis), while invasive examinations (right ventriculography and endomyocardial biopsy) are recommended only for individuals with high risk of the disease.^1^^,^^5^

The tissue criteria used in TFC 2010, obtained by endomyocardial biopsy, focused on the severity of myocyte loss and the quantification of fibrosis.^21^ However, endomyocardial biopsy is invasive and its diagnostic sensitivity may be limited due to the heterogeneous and variable distribution of the disease. Although RV free wall is often affected, biopsy is usually performed on the septum due to the fear of perforation, which further compromises its sensitivity.^22^ Rarely, outside the US, its value in the diagnosis of ARVC/D lies mainly on the differential diagnosis with other cardiomyopathies, myocarditis and sarcoidosis.^4^

### Electrocardiogram

#### Sinus rhythm

**Figure 1:**
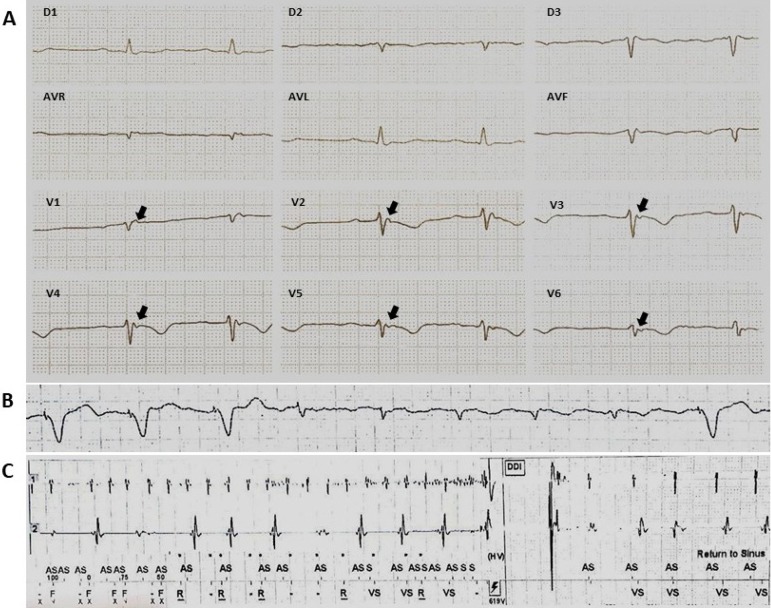
Evolution example of ARVC/D. Patient diagnosed with ARVC/D at age 32, after recovery from SAD during sports practice. He underwent implantation of ventricular ICD with multiple episodes of VF in clinical progression. At age 50, he developed sinus dysfunction and episodes of atrial fibrillation with a need for exchange for bicameral ICD. A) 12-lead ECG at diagnosis. Presence of T-wave inversion of V1-V6. Epsilon wave present in all precordial leads and final duration of QRS ≥ 55 ms. B) ECG with atrial fibrillation. C) inappropriate therapy due to atrial fibrillation.

The 12-lead ECG usually presents abnormalities in most patients with ARVC/D, indicating that electrocardiographic changes may precede the development of malignant ventricular arrhythmias (Figure 1). Thus, knowing the common manifestations of ARVC/D in the 12-lead ECG, the exercise test, and the 24-hour Holter test may be useful in increasing the diagnostic accuracy when generating the clinical suspicion.^7^ In addition, it can help with the identification of relatives affected.^6^^,^^9^ However, although ECG analysis is crucial to initial stratification, about 12% of patients with ARVC/D may present with a normal ECG, which reinforces the need for clinical evaluation that is based on the criteria proposed by TFC 2010.^9^

In addition to electrocardiographic changes classically described in ARVC/D, other alterations can be identified in the baseline ECG: sinus bradycardia, P wave abnormalities (secondary to atrial involvement), and AV conduction disorder (more often first-degree AVB). The occurrence of severe atrioventricular conduction disturbance in ARVC/D is rare.^9^

Several multicenter studies have shown that T-wave inversion in V1-3 is the most common ECG finding in ARVC/D. As a result, in TFC 2010, this T wave alteration was considered a major criterion for its diagnosis. The presence of inversion of the T wave only in V1 and V2 is a minor criterion. The inversion of T is secondary to the structural alterations of the RV. The observation of inversion beyond V3 translates a very advanced stage of the disease with severe RV dilation and possible LV involvement and can therefore be considered as indicative of worse prognosis (Figure 1A).^5^^,^^6^^,^^9^

One of the common findings of ARVC/D is complete or incomplete right bundle branch block (RBBB), especially in patients with severe structural impairment, and its presence may compromise the interpretation of ventricular depolarization abnormalities.^6^ RBBB in ARVC/D may have the following characteristics: (1) low amplitude of R wave and QRS in V1-2; (2) low R'/S ratio in V1-2; (3) inversion of the T wave in V1-3 or in the other leads of the frontal plane. Epicardial and histopathological mapping studies have demonstrated that RBBB in ARVC/D is not due to a proximal right bundle branch block, but represents the result of the distal changes inherent in delayed stimulus propagation in the regions of fibrous-fatty transformation.^9^

Epsilon wave, a low-frequency deflection that occurs at the end of the QRS and before the T wave (Figure 1), although uncommon, is a sign of the presence of an advanced stage of ARVC/D.^1^^,^^2^^,^^23^ It reflects the presence of large late potentials on the surface ECG. Although considered a major criterion for the diagnosis of ARVC/D, LPs may also be present in other pathologies, particularly cardiac sarcoidosis.^9^ These activation delays are best diagnosed with ECG-HR. Currently, a positive ECG-HR is considered a minor criterion.

Also included as a diagnostic criterion was the detection of a final activation delay, which is defined as prolongation of QRS duration (> 110 ms) and S wave (≥ 55 ms) in V1-3.

#### Arrhythmias

Increased susceptibility to ventricular tachyarrhythmia and SAD is the main characteristic of ARVC/D.^1^ In general, ventricular arrhythmias, in an isolated and frequent form, or non-sustained and sustained ventricular tachycardia are associated with symptoms of palpitation, dizziness, presyncope and syncope. Due to the most common RV origin, this ventricular arrhythmia presents LBBB morphology with a variable axis depending on the affected site.^5^^,^^6^^,^^9^

Frequent ventricular ectopy recording in the 24-hour Holter (> 500 VEx/24h) is considered a minor criterion.^7^

Few studies have evaluated the frequency of supraventricular arrhythmia in ARVC/D. Although not related to mortality, the presence of atrial arrhythmia is associated with an increase in disease morbidity, and to the increase of inappropriate therapies by ICD. The incidence of atrial arrhythmia in ARVC/D varies between 14% and 24%, and atrial fibrillation is the most prevalent supraventricular arrhythmia (Figure 1B).^24^^,^^25^ The occurrence of atrial arrhythmia is particularly associated with the presence of tricuspid insufficiency, atrial involvement and significant RV dilation.^9^

#### Complementary examinations

Echocardiogram

A structural and functional evaluation is fundamental for the diagnosis of ARVC/D. Echocardiography, as a result of its accessibility, has been the image exam of choice for the beginning of the investigation of ARVC/D. However, the unique geometry and complex pattern of RV contraction, together with the increased recognition that structural abnormalities may not be apparent in the earlier stages of the disease, limit its diagnostic utility.^7^ Echocardiographic findings suggestive of ARVC/D include: (1) global or segmental abnormality of the ventricular wall in association with dilatation of the cavity (mainly right); (2) RV hypertrophy and systolic dysfunction; (3) dilatation of the RV outflow tract (diameter > 30mm).^6^

Cardiac magnetic resonance

In the last decade, CMR has emerged as the image modality of choice in the ARVC/D investigation, because it allows for a non-invasive morphological and functional evaluation, as well as for analysis of the tissue changes (fibro-fatty transformation) that characterize this pathology.^6^^,^^7^ However, misinterpretation of CMR findings is the most common reason for misdiagnosis of ARVC/D. The most common errors include inadequate diagnosis of physiological or artefactual fat infiltration, misinterpretation of normal variants of RV wall movement, and inappropriate diagnosis in cases of sarcoidosis and myocarditis.

This “pathological” connotation given to the existence of fat in the RV led to a high incidence of false positivity, especially when using the TFC criteria of 1994. 2010 TFC brought a better definition of the criteria to be sought in the CMR, leaving the use of specific protocols for fat screening in the right chamber aside.

Abnormalities of CMR in ARVC/D can be grouped into morphological and functional abnormalities (Table 2). These abnormalities were initially observed in the classically described “triangle of dysplasia”^3^ which refers to the RV entry tract, the outflow tract and the apex. However, a recent study suggests that these changes preferentially involve the subtricuspid epicardial region, the RV free basal wall and the LV lateral wall, with the RV apex and the endocardium generally spared.^6^^,^^9^

**Table 2:** Cardiac magnetic resonance imaging findings in cardiomyopathy / arrhythmogenic right ventricular dysplasia

Functional abnormalities
Regional abnormalities of RV wall movement
Focal aneurysms
RV Dilation
Diastolic/systolic dysfunction of the RV
**Morphological abnormalities**
Intramyocardial fat infiltration
Focal fibrosis
Focal decrease of RV wall thickness
Wall hypertrophy
Trabecular disarrangement
Hypertrophy of the moderating band
RVOT diameter change

RV: right ventricle; RVOT: right ventricular outflow tract.

In addition to the parameters included in the TFC 2010, there are other characteristic abnormalities of the ARVC/D that can also be visualized by the CMR. These parameters include the RV microaneurysms and the presence of an “accordion signal”, which is the focal wrinkling of the RVOT or RV free subtricuspid wall, which is more prominent during the systole. In addition, the presence of intramyocardial fat in the RV suggests ARVC/D; however, its presence is not specific and has been observed in the elderly, in chronic users of steroids, and in other cardiomyopathies.^4^^,^^5^

Although the increase in late enhancement by gadolinium (LE) has been frequently detected in patients with ARVC/D, this criterion was not incorporated in 2010 TFC due to several limitations (RV thin walls, difficulty in differentiating fat from fibrosis, and irregular impairment of the RV). Despite this, we believe that it is of diagnostic value, especially those with biventricular or left dominant forms.^4^

The increasing use of CMR is leading to the recognition that LV is more frequently changed than previously thought, leading to the expression arrhythmogenic cardiomyopathy. LV involvement is mainly located in the inferior and basal inferolateral walls, typically in the form of fat infiltration extending from the epicardium to the myocardium. These sites may also present LE, often without an association of ventricular wall motility abnormality.

Electrophysiological study

The electrophysiological study (EFS) with programmed ventricular pacing is nowadays less used in the diagnostic and therapeutic evaluation of ARVC/D.^7^ The largest multicenter study on patients with ARVC/D who received ICD implantation showed that the EFS has limited value in predicting the risk of a severe arrhythmia. In this study, the incidence of effective therapies for fatal events (VF/VFL) did not differ significantly among patients with inducible arrhythmia or not, during baseline EFS.^26^

In spite of these recent results, 2010 TFC considers that the EFS should be taken into account for the diagnosis and evaluation of patients with suspected ARVC/D (class IIa) and may also be used in the risk stratification of asymptomatic patients (class IIb).^18^

**Figure 2:**
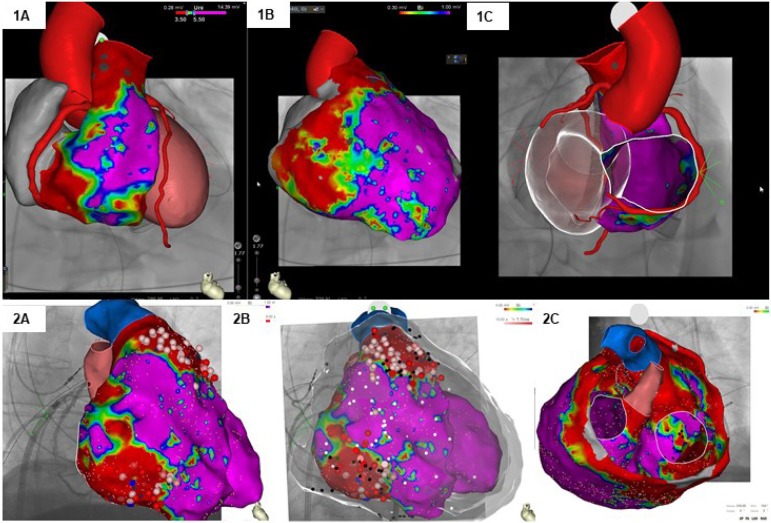
Two examples of voltage mapping for ventricular tachycardia ablation in patients with ARVC/D. 1A) Mapping of epicardial voltage showing (in red) areas of scar in the outflow tract and basal region of the RV. 1B) Mapping of endocardial voltage showing the presence of more extensive scar areas in the same region. 1C) Perspective showing the correlation of the scar areas with the coronary tree. 2A and 2B) Voltage mapping used for substrate ablation in a patient with ICD with multiple therapies. Radiofrequency applications (white and red circles) distributed in the endocardial and epicardial regions. 3C) Mapping image showing scar presence affecting the LV.

Recent studies using electroanatomic voltage mapping (bipolar and unipolar) to assess the existence and extent of the scar area in the RV have added interest in the use of EFS in the evaluation of ARVC/D (Figure 2).

This mapping technique proved to be useful in directing the region to be biopsied, as it is more sensitive than the CMR to identify myocardial scar areas, and in the differential diagnosis between an idiopathic VT of the RVOT, and a VT in a patient with ARVC/D. Nevertheless, due to the fact that it is an invasive, high cost and dependent operator, this diagnostic method should be reserved for cases with a high index of suspicion and an indefinite diagnosis.^5^

Differential diagnosis

The main differential diagnoses that should be considered in suspected cases of ARVC/D include: idiopathic RVOT VT, VT originating from the aortic cusps, and cardiac sarcoidosis.^6^

The idiopathic RVOT VT is a generally benign form of ventricular arrhythmia without association with cardiac structural alteration.^27^ The differential diagnosis is based on the fact that idiopathic VT is a non-familial arrhythmia and that the patient does not present the classic ARVC/D electrocardiographic alterations.^28^ An evaluation with the CMR should be carried out in all cases.

Another differential diagnosis is sarcoidosis. This granulomatous disease, when it involves the heart, may be very similar to ARVC/D. Cardiac sarcoidosis should be suspected when cardiac manifestations are associated with mediastinal lymphadenopathy, extracardiac sarcoidosis, especially the pulmonary one, to severe atrioventricular conduction disturbances, and the presence of a scar in the interventricular septum in the imaging evaluation.^6^ In addition, more advanced age at onset of symptoms, presence of cardiovascular comorbidities, and non-familial disease pattern should also raise suspicion of cardiac sarcoidosis.^4^ Cardiac position emission tomography may be useful for differential diagnosis.^29^

Other less frequent pathologies are: myocarditis; Brugada syndrome;^30^ dilated cardiomyopathy, in cases with biventricular dysfunction; myocardial infarction with involvement of both cardiac chambers; pulmonary hypertension (RV pressure overload), and/or significant tricuspid regurgitation (RV volume overload); congenital heart defects such as Uhl's anomaly and corrected Fallot's tetralogy; and left-right intracardiac *shunts* (usually interatrial septal defect and anomalous drainage of the pulmonary veins) that may cause right ventricular overload.

Recently, there has been much discussion of the phenotypic overlap between ARVC/D and Brugada syndrome.^30^ The ultrastructural changes that result from mutations in the desmosomes may explain this observation. From the clinical point of view, both conditions may manifest as abnormalities in the ventricular repolarization in right precordial leads, right bundle branch conduction disorder, and ventricular arrhythmias stemming from the RV.^31^ Pathologically, fatty myocardial infiltration has been reported in both conditions.^4^^,^^32^^,^^33^ As a consequence, ARVC/D and Brugada syndrome may be part of a subgroup of structural myopathies due to changes in the sodium current, due to the involvement of the inter-cellular connection.^30^

### Risk stratification

The natural history of ARVC/D is predominantly related to electrical instability that can lead to arrhythmic SAD, especially in young athletes. At a later stage of the disease, progressive RV impairment and left ventricular involvement may result in right and/or left failure.^1^^,^^6^

Data regarding the clinical progress come from small cohorts performed in tertiary centers and with a relatively short clinical follow-up. The total mortality estimated in these studies ranges from 0.08% to 3.6% per year. In community studies, which provide real-world data, annual mortality is < 1%.^4^^,^^5^

**Figure 3:**
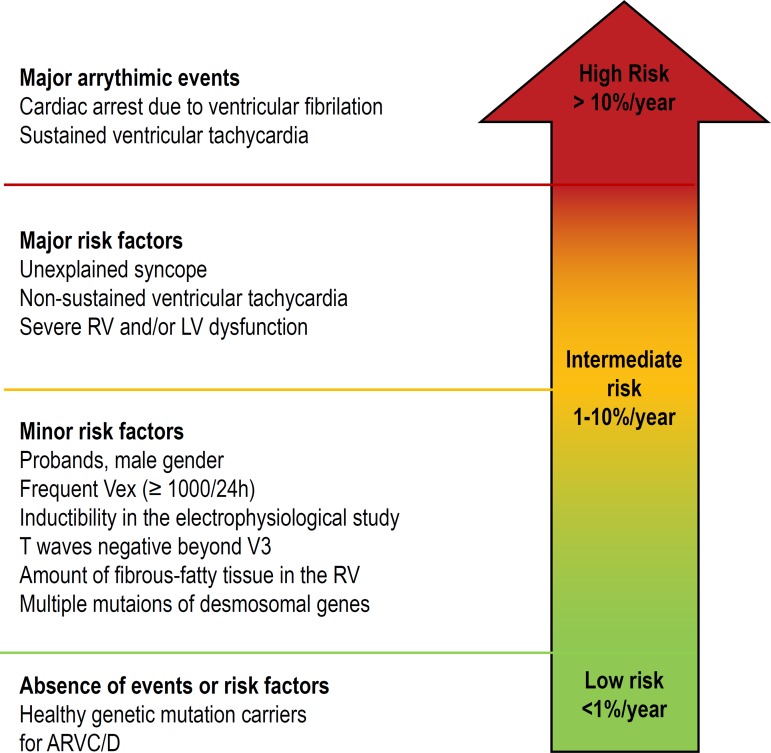
Proposed scheme for the prognostic stratification of patients with ARVC/D, according to the clinical presentation. The risk subgroups shown in the figure were defined based on the estimated probability of a major arrhythmic event (sudden cardiac death, cardiac arrest due to ventricular fibrillation, ventricular tachycardia or an event requiring ICD intervention) during follow-up, in relation to arrhythmic events or previous risk factors. An estimated annual risk of more than 10% defines the high-risk group; a risk between 1% and 10% defines the intermediate risk group; and a risk below 1% defines the low-risk group. VEx: ventricular extrasystoles; ARVC/D: cardiomyopathy/right ventricular arrhythmogenic dysplasia. Adapted from Corrado et al., 2017.^5^

Several factors were proposed for stratification of mortality risk and / or ventricular tachyarrhythmias in the ARVC/D. Corrado et al.^34^ developed a risk stratification categorized as high, intermediate and low risk. Thus, the authors sought to facilitate the early recognition of individuals who would benefit from ICD implantation (Figure 3).^4^^,^^34^

The main clinical variables considered as independent predictors of worse evolutionary prognosis are: arrhythmic malignant events (SAD, cardiac arrest due to VF, appropriate intervention of ICD, or therapy of ICD for fast VT/VF); heart transplantation; and in some studies, unexplained syncope.^5^

Other criteria, such as the result of genetic mapping and the invasive electrophysiological study, are still controversial in the literature.^5^

### Treatment

The most important goals of treating patients with ARVC/D include:

- Reduction in mortality from arrhythmic SAD or death from heart failure.
- Prevention of disease progression with consequent RV, LV or biventricular dysfunction and heart failure.
- Improvement of symptoms and quality of life by means of reduction/abolition of palpitations, VT relapses, or discharges from ICD (appropriate or inappropriate).
- Limitation of symptoms of heart failure and increased functional capacity.

Therapeutic options consist of lifestyle changes, pharmacological treatment, catheter ablation, ICD, and cardiac transplantation.^1^^,^^6^^,^^7^ Available evidence indicates that family members with a negative phenotype (carriers of healthy genes or with an unknown genotype) do not require any specific treatment other than sports restriction.^5^

#### Lifestyle change

Competitive sports activity increases the risk of SAD by two to five times in adolescents and young adults with ARVC/D.^25^

In a recent study, Ruwald et al. have established a link between SAD and intense effort in young individuals with ARVC/D. The authors followed 108 probands and demonstrated that competitive sports practice is associated with a significant increase in the VT/death combined outcome, and early phenotypic manifestation when compared to the inactivity of sedentary patients, or to the practice of recreational sports.^15^ Another finding was that the earliest start of competitive sports is associated with the early onset of clinical symptomatology.^15^

Early identification, prior to the symptomatic phase, of athletes affected by preparatory screening for the onset of physical activity and their disqualification from competitive sports activity may “save lives” (Italian experience).^34^

It is postulated that myocyte intercellular adhesion impairment can lead to tissue and organ vulnerability with consequent death of myocytes, especially during mechanical stress that occurs during competitive sports activity.^34^ Since the RV is a cardiac chamber with greater compliance than the LV, particularly during physical exercise, it becomes more susceptible to injuries, resulting in inflammation, fibrosis and, as a consequence, arrhythmias.^7^

Based on this, ITF recommends that patients with a definitive diagnosis of ARVC/D do not participate in competitive or resistance sports (class I), and may only participate in low intensity recreational sports (class IIa). The same restrictions can be applied to relatives with negative phenotype, even those that do not carry genetic mutations or with unseen genotype (class IIb).^35^

#### Pharmacological treatment

The pharmacological treatment of ARVC/D consists of the use of antiarrhythmic drugs, beta-blockers and drugs used in the treatment of heart failure.^1^

#### Antiarrhythmic therapy

The goal of antiarrhythmic treatment in ARVC/D is to prevent arrhythmic events. Literature data suggest that antiarrhythmic drugs are ineffective in preventing the occurrence of severe tachyarrhythmias in high-risk patients with ICD.^6^ Thus, antiarrhythmic therapy should be indicated as adjunctive therapy to ICD in patients with multiple appropriate therapies (class I), and may also be considered in those patients with frequent ectopic activity and/or NSVT (class IIa). In patients not having ICD and with hemodynamically tolerated VT, combined ablation/antiarrhythmic therapy may be applied (class IIb). On the other hand, the use of antiarrhythmic drugs should not be considered in asymptomatic carriers of genetic mutation and without documented ventricular arrhythmia (class III).

Amiodarone alone or in combination with beta-blockers (because it combines the synergistic effects of class III antiarrhythmic and beta-adrenergic blockade properties) is the most commonly used therapeutic regimen for the treatment of ARVC/D.^36^ Sotalol is a good therapeutic alternative, given the side effects resulting from the chronic use of amiodarone, particularly in the younger population.^7^

Although not available in our country, flecainide, when associated with a beta-blocker, may be an effective antiarrhythmic strategy of control in patients that are refractory to treatment with amiodarone or sotalol and/or catheter ablation.^37^

#### Betablockers

The ventricular arrhythmia in the ARVC/D often manifests itself in a situation of increased sympathetic tone. The current consensus is that beta-blocker therapy should be empirically instituted in all patients with a clinical diagnosis of ARVC/D.^5^^,^^7^ In contrast, there is no indication of prophylactic use of beta-blockers in healthy carriers of ARVC/D.^34^

#### Other drugs

Preload reduction drug therapy (usually diuretics and nitrates) is not yet part of the regular therapeutic arsenal of ARVC/D patients.^33^

Angiotensin converting enzyme inhibitors (ACE inhibitors) and angiotensin II receptor blockers have been advocated in patients with ARVC/D, especially in patients with evidence of structural impairment, although there are no studies demonstrating this indication in this specific condition.^6^

Continuous oral anticoagulant use is indicated for secondary prevention in patients with documented intracavitary thrombus, atrial flutter or fibrillation type arrhythmias, or with a history of thromboembolic event.^5^^,^^34^

#### Catheter ablation

VT catheter ablation is a therapeutic option for patients with continuing VT, or appropriate ICD shocks, despite optimal pharmacological therapy, including the use of amiodarone (Figure 2).^1^^,^^4^^,^^5^^,^^38^^,^^41^

Long-term VT relapses have been attributed to the progressive nature of ARVC, which leads to the development of multiple arrhythmogenic foci over time. The epicardial location of many TV reentry circuits, which reflects the propensity of the ARVC lesions to originate and progress from the epicardium, may also explain the failure of conventional endocardial mapping and catheter-only endocardial ablation.^1^^,^^2^

The increasing understanding of the arrhythmogenic substrate and the possibility of an epicardial approach allowed the observation of a significant increase in the success rate of catheter ablation in the treatment of VT in ARVC/D in recent years.^27^^,^^28^

The advent of three-dimensional (3D) navigation systems has enabled a significant advance in VT ablation in ARVC/D patients. This technique allows the mapping of the endocardial and epicardial substrate using a colored tissue voltage map, particularly in the areas that are adjacent to the tricuspid valve region and the RVOT (Figure 2).^27^^,^^28^ Based on this latest experience, ITF proposed that, in cases of unsuccessful endocardial approach, the epicardial approach should be attempted. It also recommends an endo/epi approach, as an initial strategy, in services with experience with this type of technique.^28^

The technique used for ablation depends on the patient's hemodynamic response during tachycardia. In cases of well-tolerated VT, the electrophysiological mapping and activation mapping techniques with the 3D system are the most commonly used. In the case of a VT with hemodynamic instability, the treatment consists of modifying the arrhythmogenic substrate, with ablation being done on the possible channels between areas with different voltages in combination with the elimination of fractional endocardial and epicardial signals (Figure 2).^27^^,^^28^

#### Implantable cardioverter-defibrillator

CDI implantation is the most accepted therapeutic strategy for ARVC/D patients, because the natural history of this pathology is characterized mainly by the risk of SAD, and only secondarily by contractile dysfunction that leads to progressive heart failure.^1^ Although there are no prospective randomized studies, observational studies of large registries have shown that the implantation of an ICD increases patients' survival. These studies have shown that 48-78% of patients receive appropriate ICD therapy during long-term follow-up.^4^^,^^6^^,^^29^

An observational study evaluated the clinical impact of ICD in the natural history of ARVC/D patients. At an average follow-up of 3.3 years, 24% of the patients had as an arrhythmic manifestation an episode of VF/VFL that would have been fatal in the absence of an ICD.^26^

Despite these results, it is important to note that the survival benefit with ICD is obtained at the expense of a high prosthesis cost, and a significant rate of complications during follow-up, mainly related to the occurrence of inappropriate therapies around 4%/year and changes in the electrodes.^33^ Inappropriate interventions occur between 10% and 25% of the patients, mainly in young patients and usually due to sinus tachycardia or atrial tachyarrhythmia (Figure 1C). The high rate of adverse events related to the electrodes can be explained by the peculiar pathophysiology of ARVC/D that leads to progressive loss of myocardium and to fibrous and fatty replacement that can both generate difficulties in locating a suitable place to implant the leads, and affect the thresholds of command and sensitivity during clinical follow-up.^4^^,^^5^ Another aspect is that it became evident that ICDs may be inappropriately implanted in patients with a false diagnosis of ARVC/D based on misinterpretation of CMR studies.^4^^,^^5^

Unicameral ICDs are recommended to minimize the risk of complications related to prolonged use of this device, especially in young patients. Although the number of inadequate interventions can be reduced by a dual chamber detection system, the additional lead predisposes to a greater risk of short-and long-term complications.^22^ Anti-tachycardia pacing is highly successful in terminating ventricular arrhythmia, and should be programmed into all devices.^42^ The role of the subcutaneous ICD is under investigation.

**Figure 4:**
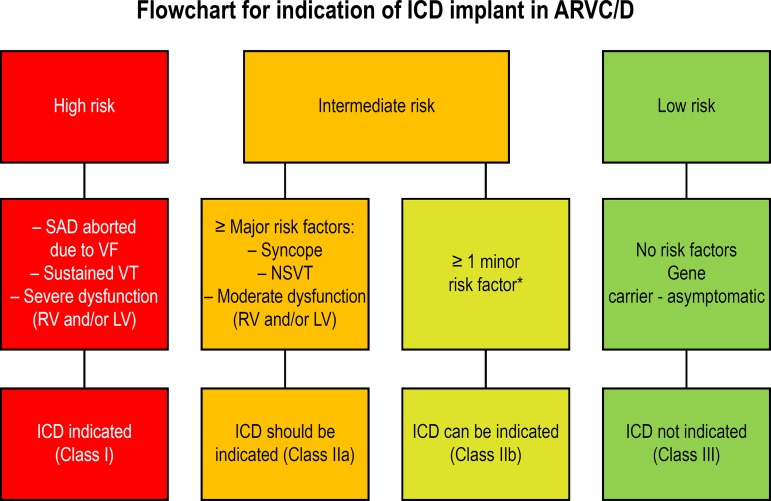
Flowchart of indications for implantation of ICD in ARVC/D. The flowchart is based on available data on annual mortality rates associated with specific risk factors. High risk of major arrhythmic events: > 10%/year; intermediate risk: 1% to 10%/year and low risk: < 1%/year. The indications for ICD implantation were determined by consensus, taking not only the statistical risk into account, but also the general health status, socioeconomic factors, psychological impact and adverse effects of the device. SCD: sudden cardiac death; VF: ventricular fibrillation; VT: ventricular tachycardia; RV: right ventricle; LV: left ventricle. *See the text for the distinction between major and minor risk factors. Adapted from Corrado et al., 2017.^22^

Based on the results of studies that defined independent predictors of major arrhythmic events (i.e. SAD, cardiac arrest due to VF, sustained VT and appropriate ICD interventions), ITF proposed an ICD indication flowchart based on three categories (Figure 4).^4^^,^^5^^,^^34^ The recommendations for the implantation of the ICD for each risk category are based not only on the statistical risk, but also on the general health, socioeconomic, psychological and adverse factors of the device.

#### Heart transplant

It is rare for a ARVC/D patient to require a heart transplant. Transplantation would be indicated as final therapy in cases of severe heart failure, and when not responsive to pharmacological treatment and resynchronization therapy (in those patients with significant LV involvement), or in patients with intractable arrhythmias (eg, incessant VT, or VF storms refractory to catheter ablation and ICD therapy).^4^^,^^5^

#### Prevention of progression

The last aspect to be considered in patients with ARVC/D is the prevention of disease progression. It is important to note that no study examined aspects that signal the evolutionary characteristics and the rate of progression of ARVC/D. Progression is slow but steady. It is suggested that the restriction of physical exercise may interfere with the rate of disease progression.^15^ A definitive curative treatment will require a deeper understanding of the biological mechanisms and environmental factors involved in the pathogenesis of ARVC/D.^5^

### Future perspectives

Significant advances were achieved if we consider the 30 years or more of the diagnosis of this pathology. However, given its rarity, many gaps persist.

It is possible to define some areas of interest that will allow better clinical management of patients and definition of the population at risk of sudden death:

- Although not yet routinely available, the future possibility of genetic screening of patients and family members with clinical suspicion of ARVC/D may become of extreme relevance with potential implications for understanding the pathogenesis and management of affected individuals.
- Further refinement in the detection of morphological abnormalities will allow a greater refinement in the algorithm for the identification of ARVC/D carriers and a better understanding of their natural history. An improvement in imaging techniques (magnetic resonance imaging and echocardiography), and the possibility of MR follow-up in patients who received MR-compatible ICD implantation.
- Studies that try to analyze the phenotype-genotype correlation may clarify the natural history of the disease, and the greater propensity for the development of malignant arrhythmias and, therefore, define the best time to initiate a medical intervention.

The denomination of this cardiomyopathy has been discussed for years. The debate between naming it RVAD or RVAC is the representation of two different views of its pathophysiology, degenerative process or developmental abnormality. Probably both visions are involved; although the terminology initially proposed by Fontaine - “dysplasia” - is probably questionable, this term has been used and accepted for 40 years, and it will persist, incorporated to the history and description of this cardiomyopathy.

## Tribute

Guy Fontaine died on March 7, 2018 at the age of 82.

He pioneered modern electrophysiology and cardiac arrhythmia therapy; a visionary investigator and mentor for many electrophysiologists. Fontaine began his contributions by studying the first cardiac pacemakers in the 1960s, and pioneered the study of catheter arrhythmias by introducing surgical cardiac mapping in 1972 for ablation of severe arrhythmias, WPW syndrome, and ventricular tachycardias, which allowed the recognition and study of RVAD, its main subject of research. He introduced catheter ablation with fulguration procedures, and studied 3D mapping methods thoroughly.

Guy Fontaine is the author of more than 700 manuscripts and book chapters. He received numerous international awards for his contributions and continued active until his last day of life, despite of a severe disabling illness.
